# The EVERT (effective verruca treatments) trial protocol: a randomised controlled trial to evaluate cryotherapy versus salicylic acid for the treatment of verrucae

**DOI:** 10.1186/1745-6215-11-12

**Published:** 2010-02-08

**Authors:** E Sarah Cockayne, The EVERT Trial Team

**Affiliations:** 1 York Trials Unit, Department of Health Sciences, University of York, Seebohm Rowntree Building (Area 4), York, YO10 5DD, UK

## Abstract

**Background:**

Verrucae are a common, infectious and sometimes painful problem. The optimal treatment for verrucae is unclear due to a lack of high quality randomised controlled trials. The primary objective of this study is to compare the clinical effectiveness of two common treatments for verrucae: cryotherapy using liquid nitrogen versus salicylic acid. Secondary objectives include a comparison of the cost-effectiveness of the treatments, and an investigation of time to clearance of verrucae, recurrence/clearance of verrucae at six months, patient satisfaction with treatment, pain associated with treatment, and use of painkillers for the treatments.

**Methods/Design:**

This is an open, pragmatic, multicentre, randomised controlled trial with two parallel groups: cryotherapy using liquid nitrogen delivered by a healthcare professional for a maximum of 4 treatments (treatments 2-3 weeks apart) or daily self-treatment with 50% salicylic acid for a maximum of 8 weeks. Two hundred and sixty-six patients aged 12 years and over with a verruca are being enrolled into the study. The primary outcome is complete clearance of all verrucae as observed on digital photographs taken at 12 weeks compared with baseline and assessed by an independent healthcare professional. Secondary outcomes include self-reported time to clearance of verrucae, self-reported clearance of verrucae at 6 months, cost-effectiveness of the treatments compared to one another, and patient acceptability of both treatments including possible side effects such as pain. The primary analysis will be intention to treat. It is planned that recruitment will be completed by December 2009 and results will be available by June 2010.

**Trial registration:**

Current Controlled Trials ISRCTN18994246.

## Background

Verrucae or plantar warts are a common, infectious and sometimes painful problem. Although most verrucae will spontaneously disappear without treatment many patients seek treatment to remove a verruca due to it being painful or because they are being prevented from doing sports and other activities of daily living. Two common treatments for verrucae are cryotherapy using liquid nitrogen which involves freezing the tissues at below -60C or application of a salicylic acid paste which macerates the skin. Using incidence figures from the 4th National Morbidity Survey [1], an economic decision model assessing the effectiveness and cost-effectiveness of salicylic acid and cryotherapy has estimated that almost 2 million people in England and Wales see their General Practitioner (GP) per year about cutaneous warts at a cost of at least £40 million per annum [2].

A systematic review conducted by the Cochrane Skin Group assessed the effects of different local treatments of cutaneous, non-genital warts in healthy people [3]. This review highlighted the uncertainty with respect to the optimal treatment of verrucae. There was, however, some evidence from six trials to suggest that treatment with salicylic acid was more effective than placebo/no treatment, odds ratio 3.91 (95% confidence interval (CI) 2.40 to 6.36). Many patients experience unpleasant side effects such as pain and blistering during cryotherapy treatment, yet the same review found no evidence to suggest that it is more effective than treatment with topical agents such as salicylic acid. Only two trials were identified which compared salicylic acid and/or lactic acid with cryotherapy, and they found no difference in the efficacy between the treatments (odds ratio 1.15, 95% CI 0.72 to 1.82). However, both trials were reported as low quality, due to unclear allocation concealment, inadequate blinding procedures, small sample sizes and inappropriate follow-up and analysis. There is a need, therefore, for a high quality randomised controlled trial with a cost-effectiveness analysis to ascertain which is the better approach for the treatment of verrucae.

The primary objective of the EVERT trial is to compare the clinical effectiveness of cryotherapy versus salicylic acid for the treatment of verrucae. Secondary objectives are to compare the cost-effectiveness of cryotherapy versus salicylic acid, and to investigate time to clearance of verrucae, recurrence/clearance of verrucae at six months, patient satisfaction with treatment, pain associated with treatment, and use of painkillers for the two treatments.

## Methods

### Design

This is an open, pragmatic, two-arm, multicentre, randomised controlled trial. Patients with verrucae are randomised equally to cryotherapy using liquid nitrogen delivered by a healthcare professional (a podiatrist, practice nurse or GP) or once daily self-treatment with 50% salicylic acid paste (Verrugon; William Ransom & Son Plc, Hitchin, UK). Two hundred and sixty-six patients are being enrolled into the study at recruiting sites in the UK and Southern Ireland for a period of 24 weeks, with treatment lasting a maximum of 8 or 9 weeks. The primary outcome is assessed at 12 weeks. Recruitment started in October 2006 and will continue until approximately December 2009. The study has been approved by the Trent Multi-centre Research Ethics Committee (Reference Number 04/mre04/59), the Medicines and Healthcare Products Regulatory Agency (MHRA), The Irish Medicines Board (IMB) and the ethics or National Health Service (NHS) research and development committee responsible for each site. The study is being conducted in accordance with the ethical principles in the Declaration of Helsinki and good practice guidelines on the proper conduct of research.

### Participants

Patients are eligible if they (i) have a verruca that in the opinion of the healthcare professional is suitable for treatment with both salicylic acid and cryotherapy and (ii) are aged 12 years and over. Patients are excluded if they (i) are currently in a trial evaluating other treatments for their verruca, (ii) have impaired healing e.g. due to diabetes, peripheral vascular disease or any other condition which means the patient has impaired healing, (iii) are immunosuppressed e.g. have agammaglobulinaemia, or are currently taking immunosuppressant drugs such as oral corticosteroids, (iv) are unable to give informed consent, (v) are currently on renal dialysis, (vi) have cold intolerance e.g. Raynaud's syndrome or cold urticaria, or (vii) have any of the following conditions: blood dyscrasias of unknown origin, cryoglobulinaemia, cryofibrinogenaemia, collagen and auto-immune disease.

Recruitment is taking place in both podiatry outpatient clinics (hospital-based or university podiatry school-based) and general practice at eight recruiting sites in the UK and one in Southern Ireland. To aid recruitment, a number of strategies have been adopted to increase the number of patients presenting to the clinics and GP practices e.g. the trial has been advertised at pharmacies, GP surgery waiting rooms, swimming pools, libraries and secondary schools local to the recruiting sites.

All patients, and their parent/guardian for patients under the age of 16 years, are given written information about the study and opportunity to discuss the study with the trial coordinator and/or site investigator prior to their decision whether to take part. Written informed consent is obtained from the patient, or the parent/guardian for those under 16 years, prior to being randomised. The healthcare professional at the recruiting site randomises the patient using the remote, independent York Trials Unit (University of York, UK) telephone or web-based randomisation service. Randomisation is simple, that is it is not restricted in any way, for example by stratification or blocked allocation. The allocation sequence is computer generated, with the treatment allocation being concealed from both the healthcare professional and York Trials Unit until the moment of randomisation.

### Interventions

For patients randomised to cryotherapy using liquid nitrogen, treatment is delivered by the healthcare professional according to the usual practice of each trial site. Prior to treatment, the callus surrounding the verruca(e) may be debrided (e.g. with a scalpel or file) with any haemorrhages stopped by digital pressure only. The tissue surrounding the verruca may be masked (e.g. with petroleum jelly) or left unmasked. Liquid nitrogen is then applied using a spray (method of choice if available), probe or cotton bud, until the healthcare professional is satisfied that the tissue has been frozen adequately. Silver nitrate may not be applied to the site. If necessary, after treatment the healthcare professional may pad the area surrounding the verruca e.g. with 7 mm felt cavitied padding. The patient is recommended to use painkillers as they would for a headache if the area is very painful. All patients are given a follow-up appointment at 2 weeks as a safety check and for further cryotherapy if considered necessary. Patients return up to two more times for further cryotherapy if required (up to a maximum of four treatments), with 14 to 21 days between treatments. All participants are given a follow-up appointment at 12 weeks for the primary outcome assessment.

Patients randomised to self-treatment with 50% salicylic acid are instructed by the healthcare professional how to use the salicylic acid paste at the first trial appointment. Thereafter, the acid paste is applied daily by the patient (or parent/guardian if appropriate) for a maximum of 8 weeks as per the manufacturer's instructions. All patients are given a follow-up appointment at 2 weeks as a safety check and at 12 weeks for the primary outcome assessment.

### Study evaluations

#### Primary outcome

The primary outcome is complete clearance of all verrucae at 12 weeks as observed on digital photographs taken by the healthcare professional delivering the treatment at baseline and 12 weeks and assessed by an independent healthcare professional. Blinded healthcare professional assessment of clearance is also performed at the 12 week appointment, to be used if for some reason the digital photograph is not interpretable. Clearance of verrucae is defined as the restoration of normal skin upon close inspection, as assessed by the healthcare professional. Participants who do not attend their 12 week outcome assessment appointment are asked if they are willing to take a digital photograph of their foot.

#### Secondary outcomes

Secondary outcomes are time to clearance of verrucae, clearance of verrucae at six months, pain intensity of the first treatment (on a scale of 0 to 10, where 0 is no pain and 10 is the worst pain imaginable), use of painkillers due to verruca treatment (number of days painkillers taken), and patient satisfaction with the treatment (on a 5 point scale, from 'very unhappy' to 'very happy'). All secondary outcome measures are self-reported by the patient and assessed by questionnaire (either paper or web-based according to the patient's preference), which patients are requested to complete at baseline, just after the first treatment and at 1, 3, 12 and 24 weeks. Patients are supplied with a form to return to York Trials Unit when their verruca has gone.

For the economic evaluation, data on the number of visits to healthcare professionals in relation to verruca treatment (trial investigator and any other) and cost of other verruca treatments purchased by the patient are collected in the 12 week questionnaire completed by the patient and from clinic records of attendance.

#### Further data collected

Other data collected in the questionnaires completed by the patients include pain associated with the verrucae (on a 5 point scale, from 'not at all' to 'extremely'), number of days over a two week period the patient has self-treated with salicylic acid, reasons for seeking treatment for the verruca(e), how long the patient has had the verruca(e), whether they received previous treatment for the verruca(e), willingness to receive the same treatment again if they had another verruca, if the patient had to stop treatment, reasons for this and whether they started another treatment, number of previous verrucae and age at which they occurred. Treatment details for both groups, number and type of verrucae and participant's treatment preference are collected via a questionnaire completed by the healthcare professional. Data on adverse events, self-reported by the patient (verbally and/or by questionnaire) or elicited by the healthcare professional following questioning of the patient at each study visit, are collected.

#### Sample size

The Cochrane systematic review [3] found only one small trial directly comparing the effectiveness of a chemical treatment, salicylic acid, with cryotherapy in patients with warts on their feet alone [4]. This poor quality study found a 58% cure rate among the patients allocated to cryotherapy compared with 41% among those treated with salicylic acid. This difference of 17% was not statistically significant. The overall cure rates from this study are smaller than those observed in two placebo controlled trials of salicylic acid, both of which reported cure rates of 85% for active treatment, possibly because more resistant verrucae were included in the study comparing cryotherapy with salicylic acid. The EVERT trial is powered to show a 15% difference in effectiveness. Sufficient patients will be recruited to give 80% power (5% two sided significance) to show a difference in cure rates of 70% versus 85% at 12 weeks. This requires 120 patients in each group; after allowing for a 10% drop-out rate, 133 will be required in each treatment group (i.e. 266 in total).

### Statistical analysis

Data on baseline demographics such as gender, age, type and duration of verrucae, and previous treatments will be summarised and descriptive summary statistics provided. For variables with continuous measures we will report the mean and standard deviation. For categorical data we will report numbers and percent.

Analysis will be by 'intention to treat'. All patients will be included in their initially randomised groups whether or not they received their allocated treatment. Analyses will be conducted blind to group. There will be a single primary analysis at the end of the study using 5% two sided significance tests. The primary outcome is complete clearance of all verrucae at 12 weeks. The two treatment groups will be compared using simple proportions of cure or not cured using the Chi squared test.

For the secondary outcomes stricter statistical levels of significance will be adopted (i.e. p = 0.01) to reduce the chance of type I error. Survival analysis of patient self-reported time to clearance of verrucae, censoring for loss to follow up, will be tested for using Cox regression adjusting for relevant co-variates to be defined before the analysis. The recurrence/clearance of verrucae at 6 months will be analysed in the same way as the primary outcome measure. The primary analysis will be repeated, but controlling for age, whether or not the verruca has been previously treated and type of verruca. Should numbers be sufficient, in order to examine whether mosaic verrucae respond less well to treatment than simple verrucae, the primary analysis will be repeated, but the type of verruca mosaic/simple will be included as a covariate and also an interaction term verruca type*treatment will be included [5]. As patients and healthcare professionals are not blinded to treatment, we will carry out a second, sub group analysis, assessing the influence of participant's treatment preference on treatment outcomes and the results of the cost-effectiveness analysis.

Data on side effects and pain intensity during and after treatment, use of painkillers, reasons for seeking treatment for the verruca, treatment details, patient satisfaction with treatment and number of verrucae, will be summarised and descriptive summary statistics provided. The number of patients discontinuing treatment prematurely for any reason will be summarised by treatment group and by reasons for discontinuation. The incidence of all suspected adverse treatment reactions will be summarised by treatment group.

For each outcome measure, the number of non-responders (missing data) will be calculated for each treatment group. We shall compare at each follow-up point the number and proportion of non-responders in each group and the type of non-responder e.g. proportion of males not responding in each group. Appropriate methods for dealing with missing data will be employed and sensitivity analyses performed.

### Economic analysis

The primary economic evaluation will be a cost-effectiveness analysis of the trial treatments. The evaluation will be carried out from the perspective of health services, which includes both NHS and non-NHS health related costs, over a time horizon of 12 weeks. The cost of resource use will be calculated for each trial participant using the data collected on the number of visits to healthcare professionals in relation to verruca treatment and cost of other verruca treatments purchased by the patient. Staff costs will be calculated using standard NHS costs [6]. Topical treatments will be costed using the British National Formulary [7] and manufacturer's costs where required. Patient outcome will be measured as the primary outcome i.e. complete clearance of all verrucae at 12 weeks. The incremental mean difference in costs between the two trial arms and incremental difference in patient outcome will be calculated and we will calculate an incremental cost effectiveness ratio of cost per patient cured at 12 weeks. If cryotherapy is less costly than salicylic acid and more effective or if cryotherapy is more costly but less effective, then one treatment clearly dominates the other and there is a clear choice about the treatment that is cost-effective. If non-dominance occurs we must weigh up the potential cost implications versus patient benefit to make a decision regarding cost-effectiveness. We will do this by relating the incremental mean costs between the two trial arms to the incremental mean outcome as a ratio, the incremental cost-effectiveness ratio (ICER). The ICER represents the additional cost per additional patient cured. Uncertainty regarding the cost-effectiveness analysis will be assessed using cost-effectiveness acceptability curves.

### Study organisation

The University of York is the Sponsor for the trial. The York Trials Unit at the University of York is coordinating the study, monitoring and verifying the data and will analyse the results. A Trial Steering Committee (TSC), consisting of an independent chair and two other independent members along with the lead investigator and other study collaborators, oversees the conduct of the trial. An independent Data Monitoring and Ethics Committee (DMEC), comprising of an independent statistician, podiatrist and Research Fellow has been set up to review safety and outcome data. Further details about the trial are available at the EVERT Trial Website [8].

## Competing interests

The authors declare that they have no competing interests.
